# GA101 (obinutuzumab) monocLonal Antibody as Consolidation Therapy In CLL (GALACTIC) trial: study protocol for a phase II/III randomised controlled trial

**DOI:** 10.1186/s13063-017-2107-0

**Published:** 2017-07-26

**Authors:** Jamie B. Oughton, Laura Collett, Dena R. Howard, Anna Hockaday, Talha Munir, Kathryn McMahon, Lucy McParland, Claire Dimbleby, David Phillips, Andy C. Rawstron, Peter Hillmen

**Affiliations:** 1 Clinical Trials Research Unit, Leeds Institute of Clinical Trials Research, University of Leeds, Leeds, LS2 9JT UK; 2 St James’s Institute of Oncology, St James’s University Hospital, Leeds, UK; 3 Haematological Malignancy Diagnostic Service, St James’s Institute of Oncology, St James’s University Hospital, Leeds, UK

**Keywords:** Chronic lymphocytic leukaemia (CLL), Obinutuzumab, GA-101, Consolidation, Phase II/III trial, Minimal residual disease (MRD), Randomised controlled trial (RCT)

## Abstract

**Background:**

Chronic lymphocytic leukaemia (CLL) is the most common adult leukaemia. Achieving minimal residual disease (MRD) negativity in CLL is an independent predictor of survival even with a variety of different treatment approaches and regardless of the line of therapy.

**Methods/design:**

GA101 (obinutuzumab) monocLonal Antibody as Consolidation Therapy In CLL (GALACTIC) is a seamless phase II/III, multi-centre, randomised, controlled, open, parallel-group trial for patients with CLL who have recently responded to chemotherapy. Participants will be randomised to receive either obinutuzumab (GA-101) consolidation or no treatment (as is standard). The phase II trial will assess safety and short-term efficacy in order to advise on continuation to a phase III trial. The primary objective for phase III is to assess the effect of consolidation therapy on progression-free survival (PFS). One hundred eighty-eight participants are planned to be recruited from forty research centres in the United Kingdom.

**Discussion:**

There is evidence that achieving MRD eradication with alemtuzumab consolidation is associated with improvements in survival and time to progression. This trial will assess whether obinutuzumab is safe in a consolidation setting and effective at eradicating MRD and improving PFS.

**Trial registration:**

ISRCTN, 64035629. Registered on 12 January 2015.

EudraCT, 2014-000880-42. Registered on 12 November 2014.

**Electronic supplementary material:**

The online version of this article (doi:10.1186/s13063-017-2107-0) contains supplementary material, which is available to authorized users.

## Background

Chronic lymphocytic leukaemia (CLL) is the most common adult leukaemia. The CLL8 trial [1, 2] has shown that even though almost double the number of patients receiving fludarabine, cyclophosphamide and rituximab (FCR) achieved minimal residual disease (MRD) negativity (defined as <0.01% CLL cells) compared with those receiving fludarabine and cyclophosphamide, once low-level MRD was achieved, both arms showed the same prognostic significance. This indirectly implies that the depth of the remission may be more important than the type of treatment given to attain that remission. Attainment of MRD negativity has also been demonstrated as an independent predictor of overall survival (OS) and progression-free survival (PFS) even with a variety of different treatment approaches and regardless of the line of therapy [3].

The researchers in the U.K. National Cancer Research Institute (NCRI) CLL207 phase II trial [4] assessed whether participants with low levels of disease could attain MRD negativity following consolidation therapy with alemtuzumab. The results showed good efficacy, with 39 (83%) of 47 participants who were consolidated attaining MRD negativity. Overall, 38% remained MRD-negative in the peripheral blood 6 months after therapy, which may be the true MRD-negative population because this represents the time for redistribution of the disease among various compartments following antibody treatment. After a median follow-up of 44 months, the participants who were MRD-negative 6 months after treatment had a significantly increased PFS time compared with those participants who were MRD-positive, with a median PFS at 3 years of 94.4% in MRD-negative participants compared with 60.0% in MRD-positive participants (*p* = 0.004). However, alemtuzumab had a significant toxicity profile, with 22 serious adverse event (SAEs), 4 of which were clinically unacceptable (1 pneumocystis pneumonia, 1 parainfluenza-related death and 2 Epstein-Barr virus-related transformed diseases), reported in 17 (36.2%) participants.

Obinutuzumab (GA-101) is a novel monoclonal antibody and has been shown to have greater efficacy in CLL than previous anti-CD20 antibodies [5, 6], with reports of MRD negativity when used to treat progressive CLL. In addition, because the cells targeted by obinutuzumab are B cells rather than all lymphocytes (including T cells), obinutuzumab is likely to be significantly less immunosuppressive than alemtuzumab. The study of obinutuzumab within the clinical trial setting has demonstrated a rapid response in peripheral blood lymphocytosis, with early reports of patients achieving MRD negativity with obinutuzumab [7–9].

### Rationale for therapeutic study

MRD-negative patients have a survival advantage in comparison to MRD-positive patients, regardless of the approach used to achieve MRD negativity. This is a seamless phase II/III randomised trial in which we will test whether consolidation with obinutuzumab to eradicate MRD leads to prolonged PFS.

## Methods/design

### Trial design

**Fig. 1:**
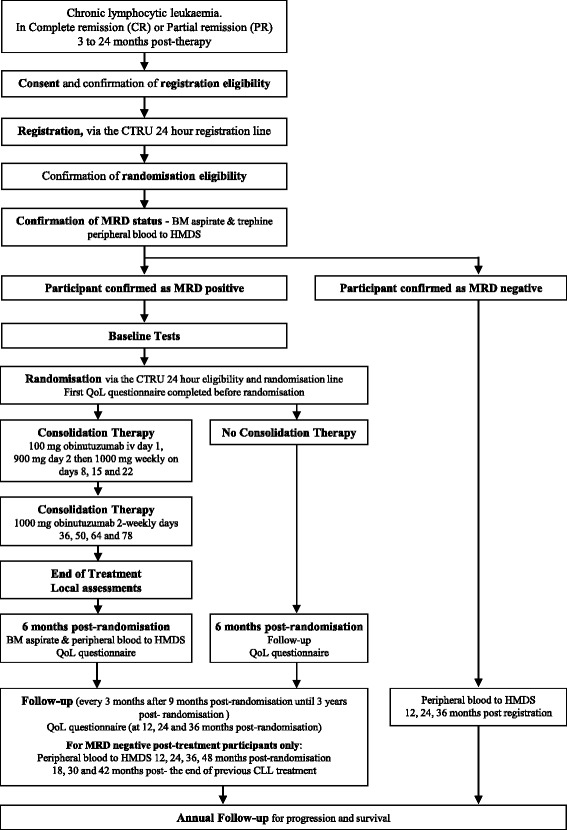
Trial flow diagram. *BM* Bone marrow, *CLL* Chronic lymphocytic leukaemia, *CTRU* Leeds Clinical Trials Research Unit, *HMDS* Haematological Malignancy Diagnostic Service, *MRD* Minimal residual disease, QoL Quality of life

**Fig. 2:**
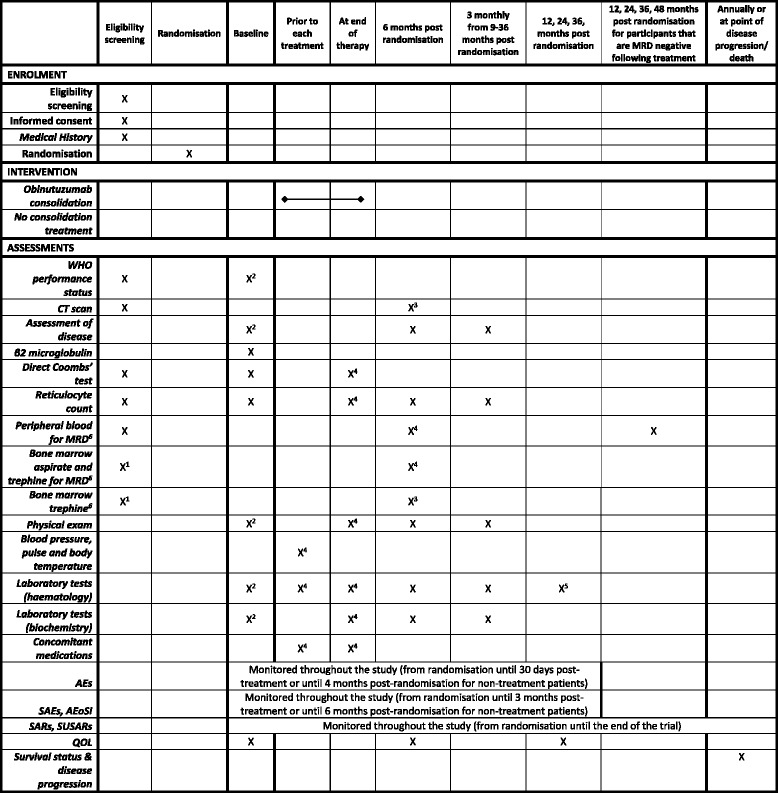
Schedule of enrolment, interventions and assessments for participants who are suitable for randomisation. *MRD* Minimal residual disease, *PFS* Progression-free survival. ^1^To be performed after the analysis of peripheral blood and only in participants whose peripheral blood is MRD positive. ^2^ To be performed within 4 weeks of randomisation and before treatment is started. ^3^Only for participants randomised to obinutuzumab and if appropriate clinically. ^4^Only required for participants randomised to treatment with obinutuzumab. ^5^Serum immunoglobulins and electrophoresis only. ^6^Tested Centrally

**Fig. 3:**
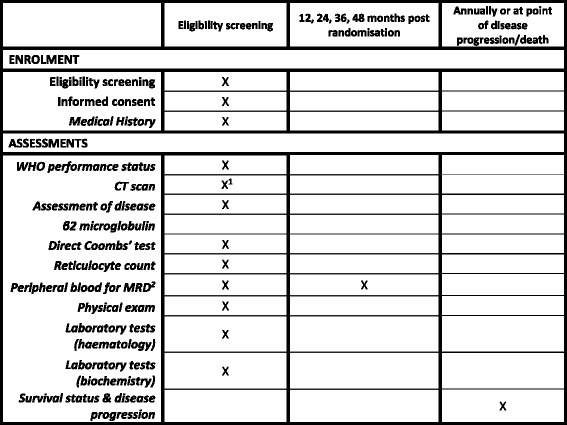
Schedule of enrolment, interventions and assessments for participants who are MRD-negative by blood analysis. *CT* Computed tomography, *MRD* Minimal residual disease, WHO World Health Organisation. ^1^If appropriate clinically. ^2^Tested Centrally

This is a seamless phase II/III, multi-centre, randomised, controlled, open, parallel-group trial for patients with CLL who have recently responded to chemotherapy. Patients with detectable MRD (MRD-positive) will be randomised to receive either consolidation therapy with obinutuzumab or no consolidation therapy (as is currently standard) (Fig. 1). The consolidation treatment period is approximately 3 months. Participants who are MRD-negative at baseline will be followed but not randomised. A schedule of enrolment, interventions and assessments is provided in Figs. 2 and 3, and a populated Standard Protocol Items: Recommendations for Interventional Trials (SPIRIT) checklist for this article is also provided (Additional file 1).

### Trial objectives

**Fig. 4:**
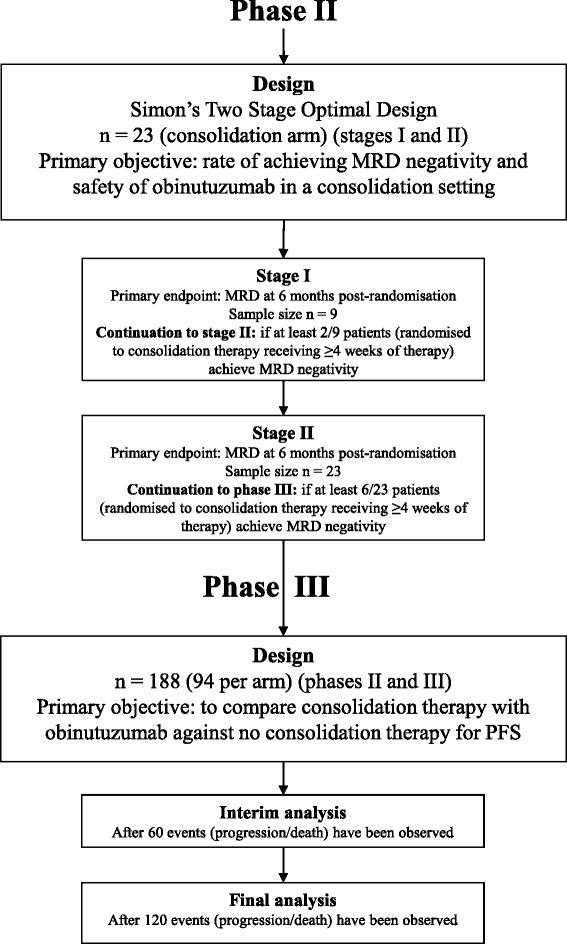
Statistical design. *CT* Computed tomography, *MRD* Minimal residual disease, WHO World Health Organisation

The trial has a seamless phase II/III design with separate primary objectives for each phase (Fig. 4). The primary objectives in phase II are to determine the rate of achieving MRD negativity at two pre-defined stages and assess the safety of obinutuzumab in a consolidation setting. The primary objective in phase III is to compare consolidation therapy with obinutuzumab against no consolidation therapy with respect to PFS in participants who responded to previous therapy with a complete remission (CR) or good partial remission (PR) with low levels of MRD.

The following are secondary objectives:

- To assess the eradication of detectable MRD, OS and time to next treatment following consolidation therapy with obinutuzumab
- To compare OS following consolidation therapy with obinutuzumab against no consolidation therapy
- To compare time to next treatment for obinutuzumab consolidation against no consolidation
- To evaluate response rate, defined as CR or PR by International Workshop on Chronic Lymphocytic Leukemia criteria [10], following consolidation therapy with obinutuzumab
- To evaluate and compare PFS and OS in participants who are and who become MRD-negative
- To assess the safety and toxicity of obinutuzumab
- To assess quality of life (QoL)

### Trial population

Patients who are eligible for the trial must be at least 18 years old, have previous confirmation of CLL, have undergone between one and three regimens of chemotherapy previously ending between 3 and 24 months before enrolment, have a peripheral B-cell count of <5 × 10^9^/L, have a response to the most recent chemotherapy treatment for CLL with PR, complete remission with incomplete marrow recovery (CRi) or CR. The patient must also be capable of giving written consent and have a World Health Organisation (WHO) performance status of 0 or 1.

Patients with any of the following characteristics are excluded from the trial: disease progression after response to last therapy, active infection, previous anaphylaxis to monoclonal antibodies, previous treatment with obinutuzumab, central nervous system involvement in CLL, mantle cell lymphoma, other severe concurrent diseases or mental disorders that could interfere with their ability to participate in the study (including moderate or worse cardiac diseases), known HIV positivity, positive serology for hepatitis B or C virus, persisting severe pancytopenia (neutrophils <0.5 × 10^9^/L or platelets <50 × 10^9^/L) or transfusion-dependent anaemia, vaccination with a live virus within 28 days, active haemolysis (patients with haemolysis controlled with prednisolone at a dose 10 mg or less per day can be entered into the trial), creatinine and bilirubin less than two times the upper limit of normal, previously treated with an allogeneic stem cell transplant, pregnant or lactating women, or women/men who are capable of conceiving children and who are unwilling to use appropriate medically approved contraception during and for 18 months after receiving treatment.

Once participants provide consent for the trial, peripheral blood will be tested for MRD. Patients who are MRD-negative will not be eligible for randomisation but will still be followed annually for MRD. Patients who are MRD-positive will have bone marrow and trephine samples tested centrally to confirm MRD level in the aspirate, a stratification factor for randomisation. To be randomised, participants must have an MRD-positive peripheral blood sample between 3 and 24 months since completing the last treatment for CLL and be confirmed as MRD-positive in the bone marrow. Participants must also have clinically and radiologically confirmed absence of lymphadenopathy (largest lymph node 1.5 cm or less in minimum diameter).

### Sample size

The overall sample size of the trial is powered for the phase III primary endpoint of PFS. An internal literature review investigating patients who have received up to three lines of previous therapy of any type and who responded to their most recent therapy with at least a PR suggested that 3 years is a reasonable estimate for median PFS in the control population (i.e., no consolidation), which translates to approximately 2 years from the time the participants are randomised (because it will be 3–24 months after previous therapy). An important clinical improvement would be an increase in median PFS from 2 to 3.3 years, a relatively large improvement, because consolidation is given at a time when patients would otherwise be in remission and treatment-free. The trial is powered to test the null hypothesis of no difference in PFS between the treatment arms, with a two-sided 5% level of significance and 80% power. Assuming 3 years of recruitment, 3 years of follow-up and allowing for a 5% drop-out rate, 188 participants are required (94 per arm) in order to observe 120 progression/death events. Losses to follow-up for the primary endpoint are expected to be extremely uncommon due to the short treatment duration and straightforward and regular follow-up schedule. A formal interim analysis on PFS will be carried out and reported to the data monitoring and ethics committee (DMEC) when half the required number of events have been observed (60 events).

The O’Brien and Fleming alpha-spending function [11] will be used to adjust for multiple testing to conserve the overall type I error, which recommends that the interim results be compared with a *p* value of 0.005, and the final analysis results are then compared with a *p* value of 0.047.

Because obinutuzumab has not previously been used in a consolidation setting, the short-term endpoint of MRD negativity will be assessed to determine whether continuation to the phase III part of the trial is worthwhile. Because it may take up to 9 months from recruiting the last participant to the time of analysis to determine their MRD response, there will be a two-stage phase II design to allow the first stage to be reviewed as early as possible and prior to the completion of recruitment to the second stage (Fig. 4). It is not planned to pause recruitment to wait for the phase II outcomes, but the DMEC, on review of the safety and early efficacy data, can choose to implement this should they feel it necessary.

Simon’s two-stage optimal design [12] will be used to set stopping bounds at each stage of the analysis (Fig. 4). A negativity rate above 35% would deem the therapy active, and a success rate below 15% would deem the therapy inactive. Participants randomised to consolidation therapy with obinutuzumab who receive at least 4 weeks of treatment will be included in the phase II populations. With a 10% significance level and 80% power, the trial will continue after the first stage if, among the first nine participants, at least two (22%) achieve MRD negativity. The trial will continue after the second stage into the phase III part of the trial if, among the first 23 participants, at least 6 (26%) achieve MRD negativity. Patients recruited during phase II will contribute to the final phase III target of 188 patients.

### Recruitment and consent

Participants will be recruited from approximately 40 research centres within the United Kingdom under the guidance of the NCRI CLL sub-group. The recruitment target requires that 188 participants be recruited into the trial, 94 in each arm.

Patients will be approached during standard clinic visits for management of CLL and provided with verbal and written details about the trial. Informed consent will be collected by principal investigators and co-investigators at the participating hospitals before any trial-specific investigations. Participants are able to withdraw from treatment and follow-up at any stage of the trial. Participants will also be approached for the UK CLL Trials Biobank, as discussed in the “Sub-studies” section below.

### Randomisation

Following confirmation of eligibility and informed consent, MRD-positive participants will be randomised into the trial by an authorised staff member at the trial research site. Randomisation will be performed centrally using the Leeds Clinical Trials Research Unit (CTRU) automated 24-hour telephone registration and randomisation system. Participants will be randomised on a 1:1 basis to receive either obinutuzumab consolidation or no treatment. Participants will be informed of their treatment allocation in this open study.

A computer-generated minimisation program that incorporates a random element will be used to ensure treatment groups are well-balanced for the following participant characteristics: bone marrow MRD level at entry (≤0.3%, >0.3%), number of lines of previous therapy (one, more than one), age group (≤65, >65 years), sex (male, female), Binet stage of disease prior to most recent CLL therapy (A progressive or B, C) and previous rituximab (yes, no).

### Protecting against bias

This trial cannot be blinded, because the two treatment arms are substantially different. Treating consultants need to be aware of the drugs being administered to be able to treat reactions to treatment accordingly. The trial researchers will use an automated randomisation service incorporating randomisation by minimisation, and analyses will be done on an intention-to-treat (ITT) basis. The trial is administered by the chief and principal investigators and the CTRU in accordance with good clinical practice (GCP) guidelines. The assessment of response in the peripheral blood and the bone marrow for MRD will be centralised in a single laboratory, thereby providing standardisation for all assessments.

### Baseline assessments

Assessments to be performed prior to the participant starting treatment are a complete physical examination, WHO performance status, serology for hepatitis B and C virus, local haematology and biochemistry, a pregnancy test (for women of childbearing potential), an assessment of disease using blood, bone marrow aspirate, trephine, computed tomographic (CT) scan and QoL questionnaires. *See* the schedule of enrolment, interventions and assessments in Figs. 2 and 3. Some of these assessments will have already been carried out to confirm eligibility for randomisation. Where these assessments are done within 4 weeks of starting treatment, the eligibility assessments will be used for baseline. Where they fall outside this time frame, they will be repeated for baseline.

### Intervention

Participants randomised to obinutuzumab consolidation will receive 100 mg of intravenous (IV) obinutuzumab on day 1 and 900 mg on day 2. The remaining course of treatment will be 1000 mg IV on days 8, 15, 22 (weekly), 36, 50, 64 and 78 (2-weekly). Participants randomised to no consolidation therapy will receive no treatment.

Participants will be assessed for their suitability for treatment within 1 week before each obinutuzumab treatment. At minimum, tests will be performed to ensure that dose delays or reductions are implemented and that concomitant medications are administered as per protocol.

Prior to medication, analgesic/antipyretic (equivalent to 1000 mg of paracetamol) will be administered before each treatment. Antihistamine (equivalent to 10 mg of cetirizine) will be administered prior to days 1 and 2 of treatment and for remaining treatments for participants with an infusion-related reaction (IRR) (Common Terminology Criteria for Adverse Events [CTCAE] [13] grade 1 or higher) to the previous infusion. Corticosteroid infusion (equivalent to 100 mg of prednisolone) will be administered for days 1 and 2 and for participants with a grade 3 IRR with the previous infusion or participants with a lymphocyte count >25 × 10^9^/L before treatment. No concomitant cytotoxic agents, radiotherapy or vaccination with live viruses should be given.

### Management of toxicity

#### IRRs

For grade 4 IRRs, the infusion will be stopped and therapy discontinued. For grade 3 IRRs, the infusion will be temporarily interrupted and symptoms treated. Upon resolution of grade 3 symptoms, infusion will be restarted at no more than half the rate being used at the time that the IRR occurred, and, if the participant does not experience any IRR symptoms, infusion rate escalation may be resumed at the increments and intervals appropriate for the treatment dose. For grades 1–2 IRRs, the infusion rate will be reduced and symptoms treated. Upon resolution of symptoms, the infusion will be continued, and, if the participant does not experience any IRR symptoms, infusion rate escalation may resume at the increments and intervals appropriate for the treatment dose. Treatment may be discontinued due to poor tolerability/toxicity at the discretion of the treating physician. The guidance in the next sub-sections is applied to manage toxicity.

#### Haematological toxicity

Participants who experience grade 3 thrombocytopenia (platelets <50 × 10^9^/L) should be withdrawn from obinutuzumab because this toxicity is potentially a result of obinutuzumab. Participants who experience grade 3/4 neutropenia (neutrophils <1.0/<0.5 × 10^9^/L) should be closely monitored. The use of granulocyte colony-stimulating factor is recommended to maintain the neutrophil count above 1.0 × 10^9^/L. Treatment with obinutuzumab should be delayed until neutrophils are > 1.0 × 10^9^/L.

#### Non-haematological toxicity

If hypersensitivity to obinutuzumab occurs (typically occurring after previous exposure to obinutuzumab), the infusion should be stopped and treatment permanently discontinued. Participants with a known immunoglobulin E-mediated hypersensitivity to obinutuzumab should not be treated. Obinutuzumab should not be administered in the presence of a severe infection. Prophylactic therapy aimed at preventing a recurrence of a diagnosed infection should be instituted if clinically indicated. For treatment of tumour lysis syndrome, electrolyte abnormalities should be corrected, renal function and fluid balance monitored and supportive care administered, including dialysis as indicated.

#### Progressive multifocal leukoencephalopathy (PML)

Cases of progressive multifocal leukoencephalopathy (PML) have been reported in patients treated with anti-CD20 antibodies, including obinutuzumab. Treatment with obinutuzumab should be withheld during the investigation of potential PML and permanently discontinued in the event of a confirmed diagnosis.

#### Hypotension

Hypotension may occur during obinutuzumab infusions. Therefore, withholding of antihypertensive treatments should be considered for 12 hours before the obinutuzumab infusion, during the obinutuzumab infusion and for the first hour after administration. Patients with a history of cardiac disease should be monitored closely. A participant should permanently discontinue obinutuzumab treatment in the following situations:

- For grade 4 infusion-related symptoms, patient should be withdrawn immediately, and treatment must be discontinued permanently
- For grade 3 infusion-related symptoms at re-challenge, patient must be withdrawn immediately, and treatment must be discontinued permanently
- Grade 3 or 4 cytopenia that has not resolved to grade 2 or lower and delays treatment of the next dose by 4 weeks
- Grade 2 or higher non-cytopenic toxicity that does not resolve to grade 1 or lower/baseline and delays treatment of the next dose by 4 weeks

### Follow-up

A formal assessment of disease response will be made 6 months post-randomisation (or at least 2 months after last treatment if treatment has been delayed). As shown in Figs. 2 and 3, this will require a physical examination and local laboratory tests; the collection of blood, bone marrow aspirate and trephine samples (for participants randomised to treatment, and trephine only where clinically indicated); CT scan (where clinically indicated); and assessment of QoL.

Follow-up visits to assess ongoing disease response and survival will be made every 3 months between 9 and 36 months post-randomisation. Participants who are MRD-negative post-consolidation treatment will also have blood collected at 12, 24, 36 and 48 months post-randomisation. All participants will be followed annually for survival until death. MRD blood assessments will end once a participant becomes MRD-positive.

All trial MRD testing will be carried out centrally by the Haematological Malignancy Diagnostic Service at Leeds Teaching Hospitals National Health Service (NHS) Trust using the harmonised European Research Initiative on CLL method [14] to ensure standardisation of reporting. Participants who are MRD-negative at baseline will have blood collected for MRD analysis at 12, 24 and 36 months post-registration. No further samples are collected if the participant becomes MRD-positive.

### Safety reporting

Adverse events (AEs) are any untoward medical occurrences in a participant who has been administered a medicinal product and do not necessarily have a causal relationship with treatment and are related to any dose administered of any trial treatment. These can be defined as any unintentional, unfavourable clinical sign or symptom; any new illness or disease; the deterioration of existing disease or illness; or any clinically relevant deterioration in any laboratory assessments or clinical tests. AEs will be recorded from randomisation until 30 days after the last dose of trial treatment (or 4 months post-randomisation for participants randomised to no treatment) or disease progression, and they will be evaluated for duration, intensity and causal relationship with the trial medication according to the CTCAE version 4.03.

SAEs and serious adverse reactions (SARs) are events that are fatal or life-threatening, require or prolong hospitalisation, are significantly or permanently disabling or incapacitating, constitute a congenital anomaly or a birth defect, or any other important medical event. SARs are SAEs that are deemed to be possibly related to any dose administered of any trial treatment. Suspected unexpected serious adverse reactions (SUSARs) are SARs that are not listed in the reference safety information for that medicinal product. SAEs will be collected from randomisation until 3 months after the last dose of treatment (or 6 months post-randomisation for participants randomised to no consolidation therapy). SARs and SUSARs should be reported from the start of treatment and for the duration of the trial. Several adverse events of special interest (AEoSI) are also to be collected until the participant begins the next line of therapy. AEoSI are tumour lysis syndrome (serious and non-serious), serious IRRs, serious infections and serious neutropenia.

### Data collection

Data will be collected on paper case report forms and entered into a validated trial database by CTRU. A validation check program will be incorporated into the trial database to verify the data, and discrepancy reports will be generated for resolution by the investigator. Priority validations will be incorporated into the validation programme to ensure that any discrepancies related to participant rights or safety are expedited to sites for resolution. Data will be monitored for quality and completeness by the CTRU. Missing data will be chased until received, confirmed as not available or the trial is at analysis. The CTRU/sponsor will reserve the right to intermittently conduct source data verification exercises on a sample of participants, which will be carried out by staff from the CTRU/sponsor. Source data verification will involve direct access to participant notes at the participating hospital sites and the central collection of copies of consent forms and other relevant investigation reports. Data will be held on a secure server at the University of Leeds, and paper case report forms will be stored in a locked unit, with both being accessible only by authorised trial staff.

### Statistical methods and analysis

Statistical analysis is the responsibility of the CTRU statisticians, and a final statistical analysis plan will be written and signed off before any analysis takes place. The primary endpoint of the phase II analyses will be carried out on participants randomised to obinutuzumab who receive at least 4 weeks of treatment. The phase III analyses will be conducted on the ITT population, where participants will be included according to the treatment to which they were randomised. A per-protocol analysis, where participants will be included according to the treatment they received, will be considered for the phase III primary endpoint of PFS if there are a considerable number of protocol violators. The safety population will consist of all participants according to the treatment to which they were randomised.

### Primary endpoint analyses

The phase II primary endpoint of MRD negativity in each stage of the phase II part of the trial will be assessed once 9 and 23 participants have received at least 4 weeks of obinutuzumab treatment and been followed until 6 months post-randomisation. The phase III primary endpoint of PFS will be assessed once 120 events (progressions/deaths) have been observed overall. A formal interim analysis will be carried out when 60 events have been observed overall in order to allow large differences between the trial arms to be reported early (Fig. 4). PFS is defined as the time from randomisation to first documented evidence of progression or death. Participants not having progressed or died or who are lost to follow-up at the time of the phase III analyses will be censored at the last date they were known to be alive and progression-free. For the analysis of primacy, we will use a Cox proportional hazards model [15] to compare the trial arms, adjusting for the minimisation factors, to calculate the HR and corresponding 95% CIs. The *p* value to assess for statistical significance will be compared with 0.047 to account for the formal interim analysis. In addition, median survival estimates with corresponding 95% CIs, as well as Kaplan-Meier curves [16], will be produced by trial arm.

### Secondary endpoint analyses

All secondary endpoint analyses comparing randomised participants will be assessed at the same time as the primary endpoint of PFS. The proportion of participants with undetectable MRD will be summarised (with 95% CIs) for participants randomised to consolidation therapy with obinutuzumab at 6 months post-randomisation and then at every time point at which MRD is assessed. CR, CRi and overall response (at least a PR) will be summarised (with 95% CIs) for participants randomised to consolidation therapy with obinutuzumab at 6 months post-randomisation and then at every time point at which response is assessed.

OS and treatment-free survival (TFS) (i.e., time from randomisation to next treatment or death) will both be assessed using Cox proportional hazards models to compare trials arms, adjusting for the minimisation factors. Kaplan-Meier curves, 95% CIs and median survival estimates will also be produced for both analyses by trial arm.

Participants who are registered and eligible but MRD-negative and so not randomised will also be followed for progression and death and will contribute to three secondary endpoint analyses. PFS, OS and duration of MRD negativity (i.e., time from end of most recent therapy for CLL to date of progression or death; date of death; or date of MRD relapse or death, respectively) will be assessed using Cox proportional hazards models to compare those who are MRD-negative at registration with those who become MRD-negative following consolidation therapy with obinutuzumab.

Occurrence and frequency of SUSARs, SARs, SAEs, AEoSI, AEs and treatment-related deaths will be summarised by trial arm and overall. In addition, all events will be summarised by maximum CTCAE grade reported and seriousness criteria, and all serious events will be summarised by Medical Dictionary for Regulatory Activities (MedDRA) System Organ Class, by trial arm and overall.

Mean QoL scores and 95% CIs, adjusted for the baseline score, will be calculated for all domains of the European Organisation for Research and Treatment of Cancer (EORTC) Core Quality of Life Questionnaire (QLQ-C30) and the CLL-specific module (EORTC QLQ-CLL16) for each trial arm and at each assessment time point and overall. Wilcoxon’s rank-sum tests will be used to compare the change in score for the trial arms from baseline to each time point.

### Interim reports

An independent DMEC will review the safety and ethics of the trial while participants are receiving trial treatment, reviewing unblinded safety updates at 3-monthly intervals, and more detailed unblinded reports at yearly intervals. The phase II analyses on the proportion of participants with undetectable MRD will be reviewed by the DMEC when 9 and 23 participants have received at least 4 weeks of consolidation therapy with obinutuzumab and been followed until 6 months post-randomisation.

A formal interim analysis on the phase III primary endpoint of PFS will be reviewed by the DMEC when half the required number of events have been observed (60 events). The DMEC, in light of the interim data, and of any advice or evidence they wish to request, will advise the trial steering committee (TSC) if there are any concerns or reasons that the trial should not continue or if they believe that the trial results should be shared further.

### Sub-studies

Trial participants will also be invited to take part in the UK CLL Trials Biobank at the University of Liverpool. Participants will be approached at baseline with a separate consent form (research ethics committee reference 14/NW/1014), and biological samples will be sent to the University of Liverpool.

### Trial organisation and administration

The trial was developed by the GALACTIC Trial Management Group (TMG) with the support of the UKCLL/NCRI CLL Clinical Trials Sub-Group. The trial is funded by Cancer Research UK and Roche. The trial is sponsored by the University of Leeds (R&D Office, 34 Hyde Terrace, Leeds, LS2 9LN, UK), co-ordinated by the CTRU, University of Leeds, and is registered with two trial databases (ISRCTN64035629, EudraCT 2014-000880-42). The trial will be conducted in accordance with the principles of GCP in clinical trials, as applicable under U.K. regulations, the NHS Research Governance Framework and through adherence to CTRU standard operating procedures. The CTRU/sponsor has systems in place to ensure that serious breaches of GCP or the trial protocol are identified and reported. Ethical approval has been obtained from the National Research Ethics Service Committee Yorkshire & Humber – Leeds East (reference 14/YH/1199). A sample of data will be assessed by on-site source data verification by the CTRU, and this is not independent from the sponsor. The University of Leeds will be liable for negligent harm caused by the design of the trial. No additional compensation for clinical negligence will be provided for trial participants over that which is available to NHS patients. Obinutuzumab was provided by the sponsor only for the duration of protocol treatment, and subsequent treatment will be provided as per standard care.

A core project team, a TMG, a TSC and a DMEC have been established. The independent DMEC will review the safety and ethics of the trial, as overseen by the independent TSC. The TSC will monitor trial progress and the overall direction. Three-monthly interim safety reports are presented to the DMEC, with a full review done annually. The DMEC will also review SARs and SUSARs that result in death in real time. This committee, in light of the interim data and of any advice or evidence they wish to request, will advise the TSC if there are any concerns or reasons that the trial should not continue. The results of the study will be published in peer-reviewed publications and will be presented at relevant national and international conferences. There are no plans to use professional writers for presenting the outcomes of this trial. Any protocol changes are disseminated by the CTRU to the relevant parties. A SPIRIT checklist was prepared for this article (Additional file 1). A SPIRIT diagram was prepared for this article (Figs. 2 and 3).

## Discussion

Recruitment for the GALACTIC trial has been challenging. Recruitment numbers provided during feasibility assessment were significantly higher than observed during trial recruitment. We believe this was due partly to improved first- and second-line treatments providing a more durable remission, resulting in fewer MRD-positive patients at 3–24 months post-treatment. With more recent targeted therapies, treatment now continues for much longer than the standard 6-month period (with FCR, for example). This is likely to have reduced the number of eligible patients.

Whilst acceptance of the trial by patients has been high, the prospect of returning to hospital for further treatment is seen as an additional burden for many, particularly those who would be required to travel a long way to their nearest hospital. Because the trial protocol represents an additional cost for the NHS Trust compared with standard treatment, it has been difficult to enrol centres into the study, despite wide support from investigators. The aim of the findings of the GALACTIC trial is to compare consolidation with obinutuzumab with no consolidation with respect to PFS in patients who have responded to previous therapy with a CR or good PR with low levels of MRD.

## Trial status

The trial opened to recruitment in February 2015 using protocol version 2.0 (registered 30 October 2014) and closed to recruitment in February 2017.

## Additional files

Additional file 1: SPIRIT checklist. (DOC 121 kb)

Additional file 2: List of participating centres. (PDF 153 kb)

## Abbreviations

AE: Adverse event
AEoSI: Adverse events of special interest
AR: Adverse reaction
BM: Bone marrow
CLL: Chronic lymphocytic leukaemia
CR: Complete remission
CR(i): Complete remission with incomplete marrow recovery
CT: Computed tomography
CTCAE: Common Terminology Criteria for Adverse Events
CTRU: Leeds Clinical Trials Research Unit
DMEC: Data monitoring and ethics committee
EORTC: European Organisation for Research and Treatment of Cancer
FCR: Fludarabine, cyclophosphamide and rituximab
GALACTIC: GA101 (obinutuzumab) monocLonal Antibody as Consolidation Therapy In CLL
GCP: Good clinical practice
HMDS: Haematological Malignancy Diagnostic Service
IRR: Infusion-related reaction
ITT: Intention to treat
IV: Intravenous
MRD: Minimal residual disease
NCRI: National Cancer Research Institute
NHS: National Health Service
OS: Overall survival
PFS: Progression-free survival
PML: Progressive multifocal leukoencephalopathy
PR: Partial remission
QLQ-C30: Core Quality of Life Questionnaire
QoL: Quality of life
SAE: Serious adverse event
SAR: Serious adverse reaction
SPIRIT: Standard Protocol Items: Recommendations for Interventional Trials
SUSAR: Suspected unexpected serious adverse reaction
TMG: Trial management group
TSC: Trial steering committee
WHO: World Health Organisation
