# Supplementary material for: GA101 (obinutuzumab) monocLonal Antibody as Consolidation Therapy In CLL (GALACTIC) trial: study protocol for a phase II/III randomised controlled trial
Source: Trials. 2017 Jul 26;18:353. doi: 10.1186/s13063-017-2107-0 (PMC5530563; doi:10.1186/s13063-017-2107-0)
Supplement: Supplementary file 2 — List of participating centres. (PDF 153 kb) [file 13063_2017_2107_MOESM2_ESM.pdf]

List of GALACTIC participating centres

Aberdeen Royal Infirmary
Addenbrookes Hospital
Arrowe Park Hospital
Basildon and Thurrock University Hospital
Basingstoke and North Hampshire Hospitals
Belfast City Hospital
Birmingham Heartlands Hospital
Cheltenham and Gloucestershire Hospitals
Darent Valley Hospital
Diana, Princes of Wales Hospital
Glan Clwyd Hospital
Glangwili General Hospital
Kings College Hospital
Leicester Royal Infirmary
Medway Maritime Hospital
Musgrove Park Hospital
Norfolk and Norwich Hospital
Queen's Hospital, Burton
Queen's Hospital, Romford
Raigmore Hospital
Royal Bournemouth Hospital
Royal Cornwall Hospital
Royal Hallamshire Hospital
Royal Hampshire Hospital
Royal Liverpool Hospital
Russells Hall Hospital
Salisbury Hospital
Scunthorpe Hospital
St Bartholomew's Hospital
St James’s University Hospital
Sunderland Royal Hospital
University College London Hospital
University Hospital South Manchester
University Hospital, Coventry
Western General Hospital
Worcestershire Royal Hospital
Wrexham Maelor Hospital
Ysbyty Gwynedd
